# Early continuous positive airway pressure versus surfactant therapy in preterm neonates with respiratory distress

**DOI:** 10.6026/973206300214575

**Published:** 2025-12-15

**Authors:** Priyasha Tripathi, Anshika Taiwade, Deepak K Uikey, Neeti Agarwal

**Affiliations:** 1 Department of pediatrics, Atal Bihari Vajpayee govt medical College, Vidisha, Madhya Pradesh, India

**Keywords:** Preterm, CPAP, surfactant therapy, neonatal respiratory distress, Level 2 SNCU, respiratory distress syndrome

## Abstract

Respiratory Distress Syndrome (RDS) remains a major cause of morbidity and mortality in preterm neonates, particularly in resource- limited Level 2 Special Newborn Care Units. Therefore, it is of interest to analyse outcomes of 308 preterm neonates treated with early Continuous Positive Airway Pressure (CPAP) or combined CPAP-surfactant therapy across three gestational age groups. Survival was highest with CPAP alone in moderate and late preterm neonates (31-36 weeks), reaching 84.4%-87.3%. Extremely preterm neonates (27-30 weeks) showed improved outcomes with early selective surfactant plus CPAP, though mortality remained higher due to pulmonary hemorrhage and sepsis. Early CPAP is highly effective for 31-36-week neonates, while extremely preterm infants benefit most from combined CPAP-surfactant therapy.

## Background:

Respiratory Distress Syndrome (RDS), primarily caused by surfactant deficiency, remains a significant challenge in neonatal medicine and is a leading cause of morbidity and mortality in preterm infants [1]. The immaturity of the lungs in these neonates leads to alveolar collapse, decreased functional residual capacity, and ventilation-perfusion mismatch, culminating in progressive respiratory failure. Over the past few decades, the management of RDS has been revolutionized by two key interventions: the application of Continuous Positive Airway Pressure (CPAP) and the administration of exogenous surfactant replacement therapy [2]. Early initiation of CPAP, often directly from the delivery room, has emerged as a cornerstone of non-invasive respiratory support. It helps stabilize alveoli, maintain functional residual capacity, conserve the infant's own surfactant, and reduce the need for invasive mechanical ventilation and its associated complications, such as ventilator-induced lung injury and bronchopulmonary dysplasia (BPD) [3]. Landmark clinical trials, including the COIN and SUPPORT trials, have provided robust evidence supporting early CPAP as a primary respiratory strategy, demonstrating outcomes comparable or superior to routine intubation and ventilation in certain preterm populations [1, 5]. Concurrently, surfactant replacement therapy has drastically improved survival rates, particularly in very and extremely preterm neonates. Administered via an endotracheal tube, it rapidly improves lung compliance and oxygenation. The standard approach has evolved from prophylactic administration to early selective or "rescue" therapy for infants with established RDS who fail to stabilize on CPAP. The INSURE (Intubate-Surfactant-Extubate) technique has become a common method to deliver surfactant while minimizing the duration of mechanical ventilation [6]. Therefore, it is of interest to evaluate the impact of these interventions on survival and mortality across different gestational age subgroups, thereby providing evidence to refine clinical practice in similar healthcare settings.

## Materials and Methods:

## Study design and setting:

This was a retrospective observational study conducted at the Level 2 Special Newborn Care Unit (SNCU) of Atal Bihari Vajpayee Government Medical College, Vidisha, and a regional referral center. All data were extracted from patient medical records. The study was conducted in accordance with the ethical principles of the Declaration of Helsinki, and institutional ethics committee approval was obtained prior to data collection.

## Study period and population:

The study included all preterm neonates admitted between June 1, 2022, and May 31, 2023. During this one-year period, a total of 1527 neonates were admitted to the SNCU. Of these, 412 were preterm. After applying the inclusion and exclusion criteria, a final sample of 308 preterm neonates was included in the study.

## Inclusion criteria:

[1] All inborn and out born preterm neonates with a gestational age between 27 and 36 completed weeks, admitted to the SNCU with respiratory distress.

[2] Neonates who received either early CPAP alone or a combination of at least one dose of surfactant replacement therapy and early CPAP.

## Exclusion criteria:

[1] Out born neonates who had received surfactant at another facility prior to transfer.

[2] Neonates who were referred out to a higher center for advanced care.

[3] Neonates with major congenital malformations affecting the cardiopulmonary system (e.g., congenital heart disease, pulmonary hypoplasia, tracheoesophageal fistula).

[4] Neonates with respiratory distress secondary to severe birth asphyxia, meconium aspiration syndrome, or confirmed congenital pneumonia.

## Data collection and stratification:

Data were collected from patient case files, including demographic information (gestational age, birth weight, sex) and clinical details (treatment modality, outcomes). The study population was stratified into three groups based on gestational age:

[1] Group A: 27 to 30 weeks (Extremely preterm)

[2] Group B: 31 to 33 weeks (Moderately preterm)

[3] Group C: 34 to 36 weeks (Late preterm)

## Intervention protocols:

Interventions were administered according to the unit's standard clinical protocols.

[1] Early CPAP: Bubble CPAP was initiated in the labor room or immediately upon admission to the SNCU for all neonates with signs of respiratory distress. Initial pressure was set at 5-6 cm H_2_O, with FiO_2_ titrated to maintain target oxygen saturation (SpO_2_) of 88-94%.

[2] Surfactant Replacement Therapy: Poractant alfa (Curosurf®) was administered at a dose of 80 mg/kg (equivalent to 2.5 mL/kg). A second dose was considered if needed. The INSURE (Intubate-Surfactant-Extubate) technique was used for administration. No prophylactic surfactant was given.

## Criteria for surfactant administration:

[1] Group A (27-30 weeks): Surfactant was administered within the first 6 hours of life for neonates with moderate to severe RDS, defined by a Silverman-Anderson score ≥6, FiO_2_ requirement >0.3 on CPAP, and/or chest X-ray findings consistent with hyaline membrane disease (HMD).

[2] Group B (31-33 weeks): Surfactant was administered selectively to neonates who demonstrated CPAP failure, defined as a persistent and increasing FiO_2_ requirement (>0.4) to maintain target SpO_2_, severe retractions, apnea, or radiological evidence of HMD.

## Outcome measures:

The primary outcomes were survival (discharged home), mortality (death during hospital stay), and LAMA (Left Against Medical Advice).

## Statistical analysis:

Data were entered into Microsoft Excel and analyzed using SPSS version 25.0 (IBM Corp., Armonk, NY). Descriptive statistics were used to summarize the data; frequencies and percentages were calculated for categorical variables (e.g., sex, treatment group, outcome), and mean ± standard deviation (SD) for continuous variables (e.g., birth weight). The Chi-square test or Fisher's exact test, as appropriate, was used to compare the proportions of outcomes (survival, mortality, LAMA) between different treatment groups within the gestational age strata. A p-value of < 0.05 was considered statistically significant.

## Results:

A total of 308 preterm neonates were included in the study, of whom 45 (14.6%) belonged to Group A (27-30 weeks), 105 (34.1%) to Group B (31-33 weeks), and 158 (51.3%) to Group C (34-36 weeks). Neonates in Group A had the lowest mean birth weight (1.1 ± 0.2 kg), followed by Group B (1.4 ± 0.3 kg) and Group C (1.8 ± 0.4 kg), while sex distribution was comparable across groups with a slight male predominance. In Group A, 40 neonates (88.9%) received surfactant plus CPAP (S+CPAP) and 5 (11.1%) received CPAP alone, whereas in Group B, 15 neonates (14.3%) received S+CPAP and 90 (85.7%) were managed with CPAP alone; all neonates in Group C were treated with CPAP alone (Table 1 - see PDF). Overall survival, mortality, and LAMA outcomes are summarized in Table 2 (see PDF). In Group A, the survival rate was 60.0% (27/45), mortality was 37.8% (17/45), and LAMA occurred in 2.2% (1/45); among those receiving S+CPAP, 62.5% (25/40) survived and 37.5% (15/40) died, while neonates managed with CPAP alone showed a survival rate of 40.0% (2/5), mortality of 40.0% (2/5), and LAMA in 20.0% (1/5). In Group B, overall survival was 81.0% (85/105), mortality was 15.2% (16/105), and LAMA was 3.8% (4/105); neonates treated with CPAP alone demonstrated higher survival (84.4%, 76/90) and lower mortality (11.1%, 10/90) compared with those receiving S+CPAP, who showed a survival rate of 60.0% (9/15) and mortality of 40.0% (6/15), with this difference being statistically significant (p < 0.05). In Group C, neonates managed with CPAP alone showed a survival rate of 87.3% (138/158), mortality of 9.5% (15/158), and LAMA in 3.2% (5/158) (Table 2 - see PDF). The causes of mortality varied across gestational age groups and treatment modalities (Table 3 - see PDF), with pulmonary hemorrhage (n=6) and sepsis (n=4) being the leading causes in Group A among neonates receiving S+CPAP, while intraventricular hemorrhage (IVH) contributed to deaths in both treatment subgroups; in Group B, sepsis was the most common cause of death in both S+CPAP (n=3) and CPAP-only (n=2) subgroups, followed by pulmonary hemorrhage and respiratory failure, whereas in Group C, mortality was primarily attributed to sepsis (n=8), followed by disseminated intravascular coagulation (DIC), ventilator-associated pneumonia (VAP), and necrotizing enterocolitis (NEC) (Table 3 - see PDF).

## Discussion:

This retrospective study provides valuable insights into the real-world effectiveness of early CPAP and selective surfactant therapy for preterm neonates with RDS in a Level 2 SNCU. Our findings underscore a gestational age-dependent treatment response, aligning with established pathophysiology and current international guidelines [7]. The principal finding of this study is the clear demarcation in optimal respiratory strategy based on gestational age. For late preterm (34-36 weeks) and the majority of moderate preterm (31-33 weeks) neonates, an "early CPAP-first" approach was highly effective, yielding survival rates of 87.3% and 84.4%, respectively. This supports the contemporary paradigm of using non-invasive ventilation as the primary modality to avoid intubation and its associated risks. By initiating CPAP in the delivery room or immediately upon admission, we likely stabilized the lungs, prevented alveolar collapse, and reduced the overall work of breathing, allowing many of these infants to avoid more invasive therapies [4]. In the moderate preterm group (31-33 weeks), the observation that the CPAP-only subgroup had better survival than the S+CPAP subgroup (84.4% vs. 60.0%) requires careful interpretation. This finding does not imply that surfactant is harmful; rather, it reflects a significant confounding by indication, a common bias in retrospective studies. The neonates who received surfactant were those who were already failing CPAP, indicating a more severe underlying lung disease and a higher baseline risk of mortality. Therefore, the combined therapy group represented a cohort of the sickest infants. This reinforces the selective or "rescue" surfactant strategy, where treatment is reserved for those who do not respond to initial non-invasive support, as recommended by Dunn *et al.* and the European Consensus Guidelines [6, 7]. Conversely, in extremely preterm neonates (27-30 weeks), severe surfactant deficiency is the predominant pathology, and CPAP alone may be insufficient. In our study, the vast majority of this group required and received early surfactant, and despite this intervention, they had the highest mortality rate (37.8%). This highlights the profound physiological vulnerability of this population. The high incidence of mortality from pulmonary hemorrhage and IVH in this group is consistent with literature highlighting the fragility of the germinal matrix and pulmonary capillaries in extremely premature infants [8]. While early surfactant is critical, these infants remain at high risk for comorbidities that contribute significantly to their outcomes. Our results are in line with the SUPPORT trial, which demonstrated that an early CPAP strategy was as effective as routine intubation and prophylactic surfactant, but also confirmed that a substantial proportion of extremely preterm infants on CPAP will ultimately require surfactant administration [5]. The causes of mortality identified in our study-sepsis, pulmonary hemorrhage, and IVH-are well-documented complications of prematurity [9]. Sepsis was a leading cause of death across all gestational age groups, emphasizing the critical importance of stringent infection control practices in the SNCU. The occurrence of VAP and NEC in the older preterm groups, who likely required longer periods of support, highlights the ongoing challenges of managing complications associated with prolonged hospitalization. This study has several limitations. Its retrospective, single-center design precludes the establishment of causality and limits the generalizability of the findings. The lack of randomization means that treatment decisions were based on clinical judgment, leading to the confounding by indication discussed earlier. Furthermore, the sample size in some subgroups, particularly the S+CPAP group for moderate preterms (n=15), was small, which can affect the statistical power of the comparison. Finally, we did not assess long-term outcomes, such as BPD or neurodevelopmental impairment, which are critical measures of the ultimate success of neonatal respiratory care. Despite these limitations, our study provides an important real-world snapshot of neonatal respiratory care in a Level 2 setting. It validates the efficacy of a protocol-driven, gestational age-based approach to managing RDS and highlights the life-saving role of both CPAP and surfactant when used appropriately.

## Conclusion:

An early, non-invasive respiratory support strategy centered on CPAP is highly effective for managing RDS in moderate and late preterm neonates in a Level 2 SNCU. CPAP should be the primary intervention, with surfactant reserved for cases of CPAP failurein this population. However, for extremely preterm neonates (<30 weeks gestation), where severe surfactant deficiency is paramount, a strategy combining early CPAP with prompt, selective surfactant administration is essential to improve survival.
